# Suppression of metastasis of human pancreatic cancer cells to the liver by small interfering RNA-mediated targeting of the midkine gene

**DOI:** 10.3892/ol.2013.1572

**Published:** 2013-09-12

**Authors:** LI YU, YU FAN, BAODING CHEN, YUE HU, YINA GAO, DA WEI

**Affiliations:** 1 Zhenjiang Key Laboratory of Molecular Endocrinology, Zhenjiang, Jiangsu 212001, P.R. China; 2 Cancer Institute, Affiliated Hospital of Jiangsu University, Zhenjiang, Jiangsu 212001, P.R. China; 3 Department of General Surgery, Jiangsu Cancer Hospital, Nanjing, Jiangsu 210009, P.R. China

**Keywords:** AsPC-1 cells, liver metastasis, midkine, pancreatic cancer, small interfering RNA, vascular endothelial growth factor

## Abstract

The present study aimed to ascertain whether suppression of midkine (MK) expression in pancreatic cancer cells inhibits metastasis to the liver. Human pancreatic cancer AsPC-1 cells were transfected with small interfering RNA (siRNA) targeting *MK*. siRNA against *MK* was observed to reduce the expression of MK mRNA and protein in a concentration- and time-dependent manner, and to decrease the number of migrating and tissue-penetrating cells in a concentration-dependent manner (P<0.005). Extracellular vascular endothelial growth factor (VEGF) concentrations were markedly reduced for the siRNA-transfected cells compared with those that were non-siRNA-transfected. The liver transmission rate and tumor nodule number in the animals harboring the siRNA-transfected cells were lower compared with those in the animals harboring the non-siRNA-transfected cells (P<0.005). These data indicate that metastasis of pancreatic cancer cells to the liver requires the expression of MK. The downregulation of VEGF expression is essential to the mechanism whereby suppression of MK expression constrains the metastasis of pancreatic cancer cells to the liver.

## Introduction

Approximately 90% of patients with pancreatic cancer present with a metastatic as opposed to localized form of the disease at the time of diagnosis (1). With the incidence of pancreatic cancer increasing over the last few decades, the treatment of metastases has become a challenge for oncologists. Pancreatic cancer cells metastasize mainly via the lymph nodes and by direct invasion, followed by hematogenous spread and extension through the neurilemma. Liver metastases are observed in half of patients in whom the cancer has metastasized and the prognosis for this group is extremely poor (1).

The *midkine (MK)* gene was first identified in 1998 by Takada *et al*(2) from the cDNA library for the retinoic acid-induced mouse testicular teratocarcinoma HM-1 cell line. Since then, the gene has been identified in several animal species and in humans. MK and the associated protein, pleiotrophin, are members of the heparin-binding factor family (3). MK is overexpressed in numerous cancers, including esophageal cancer, gastric cancer, colon cancer, pancreatic cancer, hepatocellular carcinoma and lung cancer, but is expressed at low concentrations or is absent in normal tissues (2). MK therefore possesses the potential to serve as a biomarker in the diagnosis, prognosis and treatment of patients with cancer. MK expression has been reported to correlate with the extent of metastasis of pancreatic cancer to the liver (4). However, further investigation is required to ascertain whether MK is essential to the mechanisms through which pancreatic cancer cells metastasize to the liver and other organs.

In the present study, *MK*-targeting small interfering RNA (siRNA) was employed to silence the expression of MK in human pancreatic cancer AsPC-1 cells. The migration and invasion of these cancer cells *in vitro* and *in vivo* was observed to be reduced as MK expression decreased. Experiments were also performed to investigate the involvement of vascular endothelial growth factor (VEGF) in the mechanism(s) through which the suppression of MK expression constrains the migratory and invasive capacities of these cells.

## Materials and methods

### Materials

Human pancreatic cancer AsPC-1 cells were acquired from the Tissue and Cell Bank of Jiangsu Cancer Hospital (Nanjing, China). The sense sequence of MK siRNA was 5′-AGGACUAGACGCCAAGCCUTT-3′ (Dharmacon, Inc., Lafayette, CO, USA). A monoclonal antibody to MK was obtained from Santa Cruz Biotechnology, Inc. (Santa Cruz, CA, USA) and TRIzol, RNase inhibitor, reverse transcriptase and *Taq* polymerase were purchased from Qiagen (Hilden, Germany). Oligofectamine 2000 was purchased from Invitrogen Life Technologies (Carlsbad, CA, USA). Male Balb/c nude mice (n=24) aged 4 weeks and weighing 14–18 g were purchased from the Shanghai Institute of Biochemistry of the Chinese Academy of Sciences (Shanghai, China) and housed in a specific pathogen-free (SPF) environment. Approval for this study was obtained from the Committee on Medical Ethics of the Affiliated Hospital of Jiangsu University (no. 2010035; Zhenjiang, Jiangsu, China).

### Cell culture and transfection procedures

The AsPC-1 cells were cultured at 37°C in RPMI-1640 medium containing 10% fetal bovine serum (FBS) in humidified air with 5% CO_2_. One day prior to the transfection, the logarithmically growing cells (1.0×10^5^) were seeded in 24-well plates (1 ml/well) and incubated overnight. The transfections were performed using Oligofectamine 2000 according to the manufacturer’s instructions. The cells were divided into the following groups: i) untreated control; ii) vector control (cells transfected with liposomes only) and iii) MK siRNA transfection. For the transfections, the cells were incubated in RPMI-1640 containing 10% FBS and liposomes alone or MK siRNA (embedded in liposomes) at varying concentrations. The cells were harvested for experiments by trypsinization.

### Measurement of MK expression

mRNA expression for MK was determined using qPCR. Total RNA was extracted from the harvested cells using TRIzol. The total RNA (1 μg) was used for the synthesis of the first cDNA strand using oligo dT (15-mer) as a primer and 2 μl cDNA was applied for PCR. The expression of GAPDH mRNA served as the internal reference. The PCR amplification was performed as previously described (5). The primers were forward, 5′-GCGCGCTACAATGCTCAGT-3′ and reverse, 5′-CCCTTCCCTTTCTTGGCTTT-3′. The FAM-labeled TaqMan probe was 5′-CATGGGTG CCCCGACGTTGC-3′-TAMRA. The PCR conditions included a pre-denaturation step at 95°C for 3 min followed by 35 cycles of denaturation at 95°C for 30 sec, annealing at 52°C for 45 sec and extension at 72°C for 45 sec, followed by a final extension at 72°C for 7 min.

MK protein expression was measured using western blotting, which was performed as described previously (6). The expression of β-actin (Sigma-Aldrich, St Louis, MO, USA) was used as a normalization control for protein loading.

### Measurement of extracellular VEGF concentration

Following the transfections or control incubations for 24 h, the media were collected and the cell numbers were determined. Subsequent to the clarification of the media by centrifugation at 4°C for 3 min at 800 × g, the VEGF concentration was determined using an enzyme-linked immunosorbent assay (ELISA) kit (R&D Systems, Minneapolis, MN, USA).

### Cell migration assay

A Transwell system (BD Biosciences, Franklin Lakes, NJ, USA) was employed to determine cell migration. This system was comprised of a polycarbonate microporous membrane (filter, 8-μm pore size) situated between an upper and a lower chamber that were coated with Matrigel™ (BD Biosciences). The system was placed in the wells of a 24-well plate. The cells were collected, washed three times with serum-free Dulbecco’s modified Eagle’s medium (DMEM), resuspended in serum-free DMEM and adjusted to 5×10^4^ cells/ml. DMEM containing 10% FBS (500 μl) was added to the lower chamber and the cell suspension (200 μl) was added to the upper chamber. The cells were then incubated in humidified air with 5% CO_2_ at 37°C for 24–48 h. The cells that remained on the upper filter were scraped off gently using a cotton swab and the inserts were washed gently with phosphate buffered saline (PBS). The cells that migrated to the lower chamber were fixed in 4% paraformaldehyde for 30 min, washed twice for 2–5 min, stained with 0.5% crystal violet for 30 min and washed three times with PBS for 3–5 min. Filters were placed onto the slides, which were then secured with a coverslip. Five fields were randomly selected for viewing at a magnification of ×200 and the cell number was determined. Cell migration (%) was expressed as the number of migrating cells divided by the total number of cells. Each experiment was performed three times and the results were averaged for the statistical analysis.

### Cell invasion assay

The Transwell system that was described previously was also used to measure cell invasion. The wraps were removed and the system was held at room temperature. Serum-free medium (0.5 ml) was added to the upper and lower chambers and incubated at 37°C for 2 h, following which, the medium was removed carefully. A 2.5×10^4^/ml single-cell suspension was prepared in serum-free medium and 500 μl of this suspension was placed in the upper chamber. An additional 500 μl medium containing 10% FBS was added to the lower chamber to serve as a chemoattractant. The invasion chambers were placed in bubble-free wells of plates and incubated at 37°C for 48 h in humidified air with 5% CO_2_. All the steps subsequent to the incubation were identical to those described previously for the measurement of cell migration. Each experiment was performed in triplicate and the results were averaged for the statistical analysis.

### Determination of metastasis to the liver Preparation of cells for treatments

The cells were divided into three groups, untreated control, vector control (liposomes only) and MK siRNA-treated. The transfections were performed as described previously, with the exception that liposome-embedded siRNA was used at a concentration of 12.5 nmol/l. Following two days of transfection, the cells were digested with 0.25% trypsin and 0.02% EDTA and the suspensions were subjected to centrifugation at 1,500 × g for 5 min. The supernatant fluid was removed and the cells were resuspended in normal saline to 2×10^8^/ml. The percentage of viable cells was calculated following trypan blue staining and the viability was routinely ≥95%.

### Mouse model of pancreatic cancer metastasis to the liver

A mouse model of pancreatic cancer metastasis to the liver was established using the splenectomy method (5). In brief, the nude mice were intraperitoneally anesthetized with 1% sodium pentobarbital (35 mg/kg) and fixed to a table. Following sterilization, a 1-cm longitudinal incision was made in the left upper quadrant. The gastrosplenic ligament and short gastric vessels were disconnected and the spleen was exposed. The cell suspension (0.1 ml) was injected into the upper region of the spleen over a period of 1–2 min, the syringe needle was slowly removed and this was followed by kneading. The splenic pedicle was ligated, the spleen was removed and the wound was closed. A total of five nude mice were used for each treatment group. Following the surgery, the mice were housed in an SPF environment and the spirit, food intake and body weight of the animals were monitored daily. The mice were sacrificed by cervical dislocation at 28 days post-surgery and metastasis of the pancreatic cancer cells to the liver was observed. The gross tumor nodules were counted and liver cross-sections were then used to count the tumor nodules under a light microscope. The liver was then obtained, fixed in 10% neutral formaldehyde, embedded in paraffin and cut into 4-μm sections. Five sections were selected, with 2 mm serving as the distance between the two adjacent sections. The coronal section with the maximal area was set as the center and the number of tumor nodules was then determined under a microscope. The same nodule appearing in various sections was considered a single nodule. The absence of gross or microscopic tumor nodules was regarded as the absence of metastasis. The total number of tumor nodules was determined as the sum of the gross and microscopic tumor nodules.

### Measurement of intratumoral microvessel density

The microvessel densities were determined as described previously by Rydén *et al*(6). Brown endothelial cells or endothelial cell clusters with clear boundaries with adjacent microvessels, cancer cells and other tissues were counted as a vessel, but lumen-like structures or red blood cells were not considered in this measurement. Subsequent to performing immunohistochemistry to confirm the presence of CD34, three areas that were rich in microvessels were selected under a microscope at low magnifications (×40 and ×100) and the number of vessels was determined at a magnification of ×400. The results were averaged for the statistical analysis.

### Statistical analyses

The results are presented as the mean ± standard deviation. The statistical analysis was performed using the SPSS version 11.5 statistical software package (SPSS, Inc., Chicago, IL, USA). The comparisons between the groups were performed using one-way analysis of variance. Analysis of variance was used to evaluate the findings from the animal model studies. P<0.05 was considered to indicate a statistically significant difference.

## Results

### Expression of MK mRNA and protein as a function of transfection with MK siRNA

**Figure 1:**
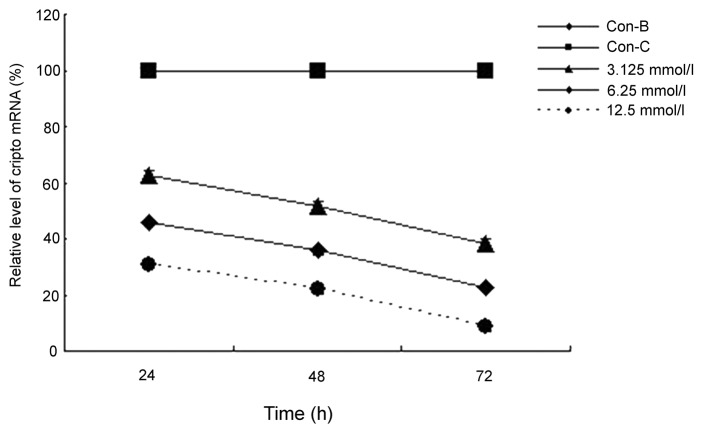
Expression of MK mRNA by human pancreatic cancer AsPC-1 cells that were transfected with siRNA against the gene encoding MK or treated with the vector only. The cells were treated with liposomes alone (Con-B) or with liposomes containing the indicated concentrations of MK siRNA. Incubations were then conducted for the times indicated, followed by measurements of MK mRNA by PCR. MK, midkine; siRNA, small interfering RNA; Con-C, untreated cells.

**Figure 2:**
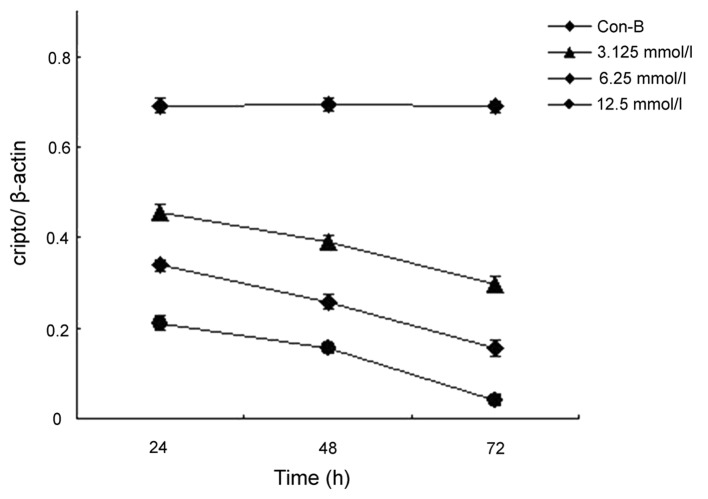
Expression of MK by AsPC-1 cells that were transfected with MK siRNA or treated with the vector only. The cells were transfected with liposomes alone (Con-B) or with liposomes containing MK siRNA at the indicated concentrations. Incubations were then conducted for the times indicated, followed by measurements of MK by western blotting. MK, midkine; siRNA, small interfering RNA.

When compared with the AsPC-1 cells that were treated with the vector only (Con-B), the cells that were transfected with MK siRNA exhibited significantly decreased levels of MK mRNA (Fig. 1) and protein (Fig. 2) expression. Statistically significant decreases were observed at 24, 48 and 72 h following the transfection procedure, and these decreases were concentration- and time-dependent (P<0.0001 and P<0.0001, respectively).

### Pancreatic cancer cell migration and invasion as a function of transfection with MK siRNA

**Figure 3:**
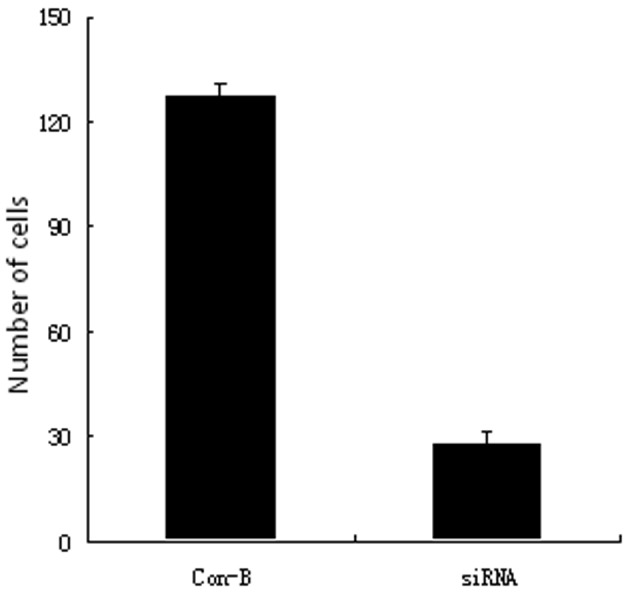
Migration of AsPC-1 cells that were transfected with MK siRNA or treated with the vector only. The cells were treated with liposomes alone (Con-B) or with liposomes containing MK siRNA. Cell migration was then determined using a Transwell system as described in Materials and methods. MK, midkine; siRNA, small interfering RNA.

**Figure 4:**
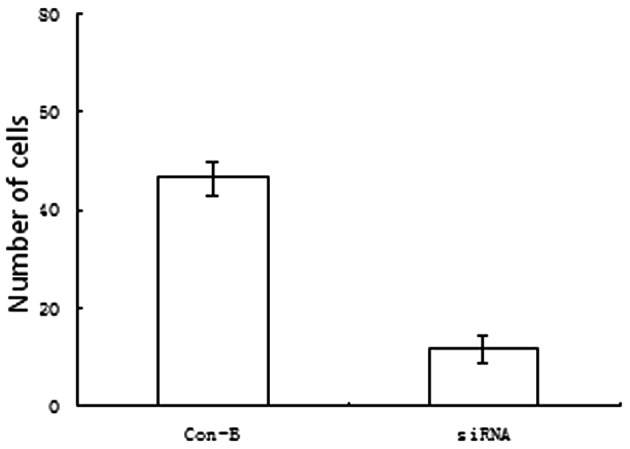
Invasion of AsPC-1 cells that were transfected with MK siRNA or treated with the vector only. The cells were treated with liposomes alone (Con B) or with liposomes containing MK siRNA. Cell invasion was then determined using a Transwell System as described in Materials and methods. MK, midkine; siRNA, small interfering RNA.

The AsPC-1 cells were harvested at 48 h following treatment with the vector only or with the vector containing MK siRNA, and a Transwell system was employed for obtaining the measurements of migration and invasion. The numbers of migrating (Fig. 3) and tissue-penetrating (Fig. 4) cells were observed to be markedly lower for the cells that were transfected with MK siRNA compared with the vector-only controls (P<0.005 and P<0.005, respectively).

### Extracellular VEGF concentrations for mock-transfected and MK siRNA-transfected AsPC-1 cells

**Figure 5:**
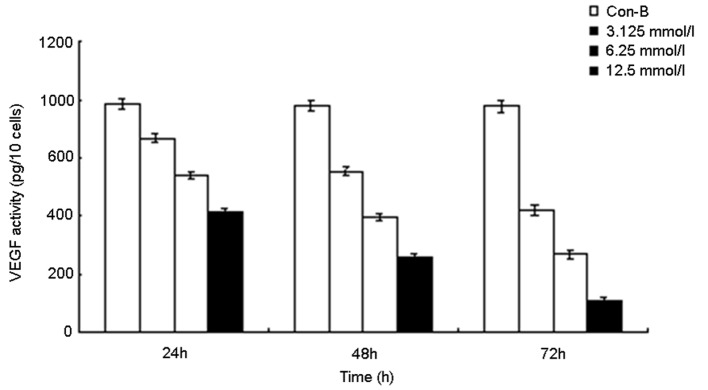
Extracellular VEGF concentrations of AsPC-1 cells that were transfected with MK siRNA at various concentrations or treated with the vector only. The cells were treated with the vector only (Con-B) or with liposomes containing the indicated concentrations of MK siRNA. Following the incubation periods of the indicated times, the medium was collected for measurement of VEGF concentration by ELISA. VEGF, vascular endothelial growth factor; MK, midkine; siRNA, small interfering RNA; ELISA, enzyme-linked immunosorbent assay.

The cells were treated with the vector only or were transfected with liposomes containing various concentrations of MK siRNA. Following 0, 24, 48 and 72 h of incubation, the medium was collected for measurement of VEGF by ELISA (Fig. 5). The VEGF concentration in the medium from the MK siRNA-transfected group was observed to decline in a manner that was dependent on the MK siRNA concentration and the time of incubation (r=0.928). By contrast, the VEGF concentration in the medium from the vector-only control group was stable throughout the 72-h incubation period.

### Effect of transfection of AsPC-1 cells with MK siRNA on liver tumor nodule number and rate of liver metastasis in vivo

A splenectomy method was employed to establish a nude mouse model of liver metastasis of pancreatic cancer cells. The livers of these animals were bright red, soft and lacked gross and microscopic tumor nodules prior to being injected with the AsPC-1 cells that had been transfected with MK siRNA (siRNA-transfected group), mock-transfected (vector only group) or left untreated (non-treated control group). However, in the mice harboring the AsPC-1 cells that had metastasized to the liver, the liver volume was decreased and the organ was hard and fragile. Furthermore, multiple gray nodules were present in the livers of these animals. The metastasis rates were 22.8±1.8, 82.6±1.6 and 81.9±1.7% for the siRNA-transfected, vector only and non-treated control groups, respectively. The tumor nodule numbers were 3.6±0.8, 18.6±1.6 and 19.1±1.5 for the siRNA-transfected, vector only and non-treated control groups, respectively. The statistical analysis revealed that the metastasis rate and the tumor nodule number for the siRNA-transfected group were significantly lower than those for the other two groups (P<0.05 and P<0.05, respectively).

### Effect of transfection of AsPC-1 cells with MK siRNA on liver tumor microvessel density in vivo

**Figure 6:**
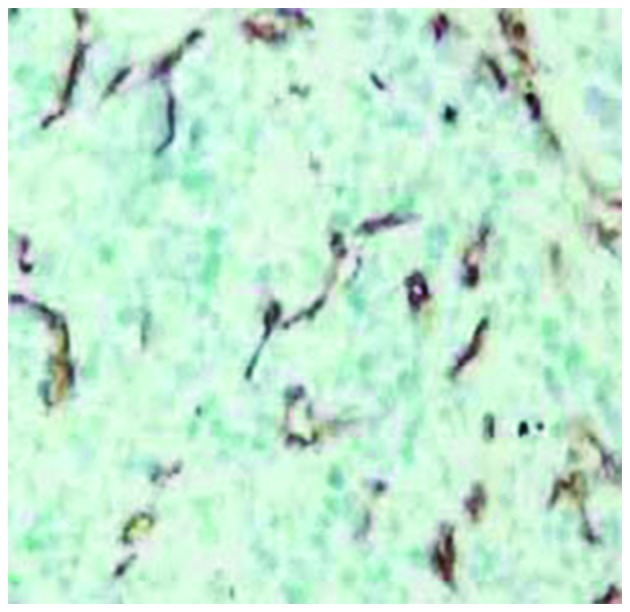
Microvessel-rich region of a liver tumor in a mouse harboring AsPC-1 cells that were treated with the vector only. Immunohistochemistry was performed to reveal the presence of CD34. The brown areas indicate microvessels (magnification, ×400; trypan blue staining).

**Figure 7:**
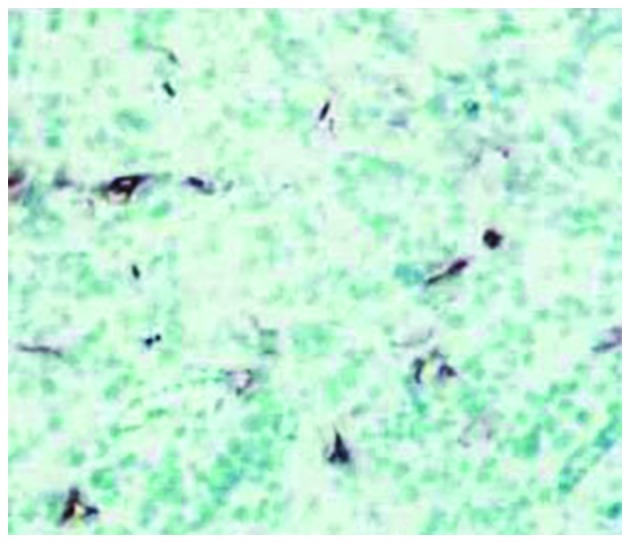
Microvessel-rich region of a liver tumor in a mouse harboring AsPC-1 cells that were transfected with MK siRNA. Immunohistochemistry was performed to reveal the presence of CD34. The brown areas indicate microvessels (magnification, ×400; trypan blue staining). MK, midkine; siRNA, small interfering RNA.

The microvessel density values were determined using the nude mouse model described previously. The value for the siRNA-transfected group was 7.56±1.68, which was significantly lower than that of the vector-only group (15.69±2.51) and for the untreated control group (16.35±2.08; P<0.05). The difference in the values between the vector-only and untreated control groups was not significant (P>0.05). The microvessel-rich areas of the liver tumors from the animals harboring the AsPC-1 cells that were treated with the vector only (Fig. 6) or transfected with MK siRNA (Fig. 7) are presented for comparison.

## Discussion

The present study revealed that the transfection of AsPC-1 cells with siRNA directed against MK is highly effective in reducing the expression of MK and its mRNA by pancreatic cancer cells. The expression was decreased in a time- and concentration-dependent manner, supporting the conclusion that RNA interference through the use of siRNA against *MK* is a suitable approach for examining the significance of MK expression in the ability of these cells to metastasize to the liver. Accordingly, transfection with MK siRNA was observed to decrease the capacities for migration and tissue penetration. Furthermore, the liver transmission rate and the number of liver tumor nodules for animals harboring the siRNA-transfected cells were reduced compared with those of the animals that harbored the non-siRNA-transfected cells. The microvessel densities of the livers from the mice that were transplanted with the siRNA-transfected cells were also significantly lower than those from the mice that were transplanted with the non-siRNA-transfected cells. Collectively, these results strongly support the conclusion that metastasis of pancreatic cancer cells to the liver requires the expression of MK by these cells.

The Transwell system has been a useful tool for studies of cellular migration and invasion *in vitro*(7,8). Cancer cells bind to a specific matrix material (Matrigel) in this system in a manner that mimics their binding to laminin, fibronectin or type IV collagen that is present in the basement membrane *in vivo*. The secretion of proteases or the activation of zymogen in the matrix by the bound cancer cells results in the degradation of the matrix. Subsequent migration of the cancer cells results in the matrix gap being filled. *In vivo*, these processes are repeated, leading to deep invasion and distant metastasis. Using the Transwell system, transfection of the AsPC-1 cells with MK siRNA was observed to decrease the number of migrating and invading cells in a manner that was dependent on the siRNA concentration, supporting a requirement for MK expression in the migratory and invasive capacities of pancreatic cancer cells.

The present study employed the splenectomy method to establish a mouse model of liver metastasis of pancreatic cancer. Although this model did not involve a splenic tumor, liver metastasis was observed. The process of metastasis using this model is similar to that observed in clinical practice, involving the spread of the cancer cells through the portal vein following pancreatic cancer resection. For the animals that were transplanted with the AsPC-1 cells that were transfected with MK siRNA, the number of metastatic liver nodules and the rate of metastasis to the liver were markedly lower compared with those in the animals that were transplanted with the control AsPC-1 cells. These observations strongly support a requirement for MK in the process through which pancreatic cancer cells spread and invade the liver.

Liver metastasis is a complex process involving multiple factors. VEGF is considered to be involved in the mechanism through which gastrointestinal cancer cells metastasize to the liver (9–11). The downregulation of VEGF is reported to suppress metastasis to the liver and the invasion of this organ by various cancers (11–14). Seo *et al* reported that VEGF is closely associated with metastasis of pancreatic cancer to the liver (11). In the present study, VEGF expression by the AsPC-1 cells was significantly decreased following their transfection with MK siRNA. Furthermore, the microvessel density of the liver tumors from the mice that were transplanted with MK siRNA-transfected AsPC-1 cells was significantly lower than that of the tumors from the mice that were transplanted with the non-siRNA-transfected AsPC-1 control cells. These findings are consistent with the hypothesis that downregulation of VEGF expression mediates the suppression of liver metastasis of pancreatic cancer cells due to repressed MK expression.

In conclusion, the expression of MK by pancreatic cancer cells is required for the metastasis of these cells to the liver. Silencing of MK expression by transfection with MK siRNA markedly decreases the capacities of cloned pancreatic cancer cells for migration and tissue penetration *in vitro* and their capacity to invade the liver *in vivo*. The downregulation of VEGF expression is likely to be involved in the mechanisms through which the reduction of MK limits the migratory and invasive properties of pancreatic cancer cells.
